# Rapid Detection of Hypervirulent Klebsiella pneumoniae by Multiplex PCR and Nucleic Acid Chromatography: Evaluation of Diagnostic Performance and Clinical Utility

**DOI:** 10.7759/cureus.106309

**Published:** 2026-04-02

**Authors:** Mieko Tokano, Norihito Tarumoto

**Affiliations:** 1 Department of Infectious Disease and Infection Control, Saitama Medical University, Moroyama, JPN

**Keywords:** dna chromatography, hypervirulent, iuca-pp2, klebsiella pneumoniae, multiplex pcr, peg-344-pp2, prmpa

## Abstract

Background and aim: Hypervirulent* Klebsiella pneumoniae* (hvKp) causes severe community-acquired infections with metastatic complications. The string test is widely used as a phenotypic marker; however, its diagnostic accuracy is limited. This study aimed to improve hypervirulent phenotype identification by combining the string test with rapid multiplex polymerase chain reaction (PCR) targeting hvKp-associated virulence genes and detection by nucleic acid chromatography.

Methods: We established a multiplex PCR and nucleic acid chromatography system capable of detecting three virulence genes (prmpA, iucA-PP2, and peg-344-PP2). We included 119 cases of bacteremia caused by isolates identified as *K. pneumoniae* by a parCgene sequence analysis. The presence of the three virulence genes was assessed using both singleplex and multiplex PCR. Multiplex PCR products were detected using agarose gel electrophoresis and nucleic acid chromatography. Clinical characteristics were compared between the hypervirulent and non-hypervirulent phenotype groups. Strains with the disseminated lesion, abscess formation, or death during the first 30 days were classified as hypervirulent phenotype.

Results: Isolates that were string test-negative but carried hvKp-associated virulence genes were identified, suggesting that reliance on phenotypic methods alone may lead to underdiagnosis of hypervirulent strains. In a receiver operating characteristic curve analysis, positivity for three or more markers among the four variables (string test and the three virulence-associated genes) yielded a sensitivity of 0.84 and specificity of 0.90. High concordance rates were also observed between singleplex and multiplex PCR using either gel electrophoresis or DNA chromatography.

Conclusions: HvKp-associated genes can be detected more rapidly by combining multiplex PCR with nucleic acid chromatography than by conventional gel electrophoresis-based methods. This approach also demonstrated high sensitivity and may be helpful for potential clinical risk assessment of hvKp infections.

## Introduction

*Klebsiella pneumoniae* is a major Enterobacterales pathogen causing a range of infections, including bloodstream infections with high mortality [1,2]. Recently, various virulence genes of* K. pneumoniae* have been reported to be associated with clinical features, microbiological characteristics (including hypermucoviscosity), and mortality rate [1]. In the 1980s, community-acquired primary liver abscesses caused by *K. pneumoniae* were reported from Taiwan [3,4]. These cases were presented at multiple sites or subsequently spread (metastatic spread). To distinguish this pathotype from classical *K. pneumoniae*, the designation hypervirulent *K. pneumoniae* (hvKp) has been used [5]. Characteristic features of the hypervirulent phenotype include a positive string test, absence of underlying biliary disease, occurrence in otherwise healthy individuals or patients with diabetes mellitus, community onset, a high frequency of metastatic infections, and a tendency toward severe disease [6,7]. Although the hypervirulent phenotype was previously observed predominantly in Asian countries, it is now widely reported worldwide [8].

Regarding the hypervirulent phenotype, which is clinically characterized by metastatic spread and high mortality rate, the hypermucoviscous phenotype of *K. pneumoniae* (string test positivity) has been shown to be associated with hypervirulence [6,9]. However, the correlation between string test results and the clinical features observed in hvKp infections is inconsistent, and the low sensitivity and specificity of the string test have been recognized as major limitations. In contrast, several genotypic biomarkers have been suggested to be useful for the diagnosis of hvKp infections [5]. According to the relevant literature, genes located on large virulence plasmids or within chromosomal pathogenicity islands are associated with the hypervirulence of hvKp strains. These include several genes carried on virulence plasmids, namely iucA (aerobactin siderophore biosynthesis), the plasmid-bornermpAgene (prmpA), which regulates the mucoid phenotype through increased capsule production, and peg-344-PP2 (a putative transporter) [5]. The rapid identification of these genes may enable early detection of complications and facilitate the timely initiation of appropriate antimicrobial therapy.

In this study, to rapidly identify virulence genes, we performed multiplex polymerase chain reaction (PCR) targeting three virulence genes specific to hvKp strains. Using a nucleic acid chromatographic method that allows for more rapid detection than conventional gel electrophoresis and staining, we assessed the presence of these virulence genes. Furthermore, we evaluated the gene positivity rates of isolates obtained from patients with the hypervirulent phenotype at our institution and analyzed their associated clinical backgrounds to assess the clinical utility of this approach.

## Materials and methods

Clinical parameters

We retrospectively collected patient information from the electronic medical records of Saitama Medical University Hospital, a 1,000-bed tertiary emergency hospital in Saitama, Japan. The data collected included the following: age, sex, patient risk factors (underlying disease, use of immunosuppressants and steroids, hemodialysis, and neutropenia; neutrophil count <500 cells/mm^3^), source of infection (which was reviewed retrospectively and described as "unknown" if not described in the medical records or determined by the attending physicians), polymicrobial bacteremia, abscess formation, dissemination, 30-day mortality, hospital-acquired infection versus community-acquired infection (infections were defined as hospital-acquired if the blood cultures were collected >48 h after admission to the hospital or if the patient had been admitted to the hospital within the previous 30 days), and appropriate or inappropriate antibiotic therapy within 24 h from the first bloodstream infection (BSI) episode. Thirty-day mortality was defined as death due to *K. pneumoniae* bacteremia. Cases in which bacteremia may have contributed to poor outcomes were considered as 30-day mortality, regardless of the primary cause of death. Appropriate antibiotic therapy was defined as the use of an antimicrobial agent to which the isolates were susceptible, as determined by in vitro susceptibility testing. The hypervirulent phenotype was defined as isolates associated with disseminated lesions, abscess formation, or death within 30 days.

Multiplex PCR

Three stored isolates classified as hypervirulent phenotypes were used. Specifically, these strains were obtained from patients admitted to Saitama Medical University Hospital in whom *K. pneumoniae* was detected in blood samples and clinically determined to have a hypervirulent phenotype. All three isolates were string test-positive. In addition, singleplex PCR confirmed that all three targeted virulence-associated genes (iucA-PP2, peg-344-PP2, and prmpA) were positive in these isolates. The string test was performed by stretching a mucoviscous string from each colony cultured on 5% sheep blood agar using a standard bacteriological loop. The formation of a viscous string ≥5 mm in length was regarded as positive, indicating a hypermucoviscous phenotype [10]. Singleplex PCR amplicons were generated using TaKaRa SPPD STA (Kusatsu, Japan: Takara Bio Inc.), and thermal cycling was carried out under the following conditions: 98°C for 2 min, followed by 40 cycles at 98°C for 5 s, 55°C for 15 s, 72°C for 10 s, with a final extension at 72°C for 3 min. Primers described in a previous report were used [5]. Specifically, the following primers were used: iucA-PP2-F (GCTTATTTCTCCCCAACCC), iucA-PP2-R (TCAGCCCTTTAGCGACAAG), peg-344-PP2-F (AAAGGACAGAAAGCCAGTG), peg-344-PP2-R (CAATGACGAGGGGGATAATC), prmpA-F (GAGTAGTTAATAAATCAATAGCAAT), and prmpA-R (CAGTAGGCATTGCAGCA).

A multiplex PCR system capable of simultaneously detecting three virulence genes was established using previously reported primers, as described above [5]. Bacterial suspensions adjusted to approximately 10^4^ colony-forming units (CFUs) and heat-treated at 95°C for 5 min were used as the PCR template. Multiplex PCR amplicons were generated using KOD Multi & Epi (Osaka, Japan: TOYOBO CO., LTD.), and thermal cycling was carried out under the following conditions: 94°C for 5 min, followed by 30 cycles at 98°C for 10 s, 55°C for 30 s, 68°C for 60 s, with a final extension at 72°C for 5 min. PCR products were analyzed using a 1% (w/v) agarose gel stained with ethidium bromide. This multiplex assay was optimized in both singleplex and multiplex formats by adjusting key PCR parameters to ensure clear and reproducible amplification of all three targets. To confirm assay accuracy, artificial DNA was used (appendix 1). The target genes (iucA-PP2, peg-344-PP2, and prmpA) were cloned into the pEX-A2J2 vector.

DNA chromatography

**Figure 1:**
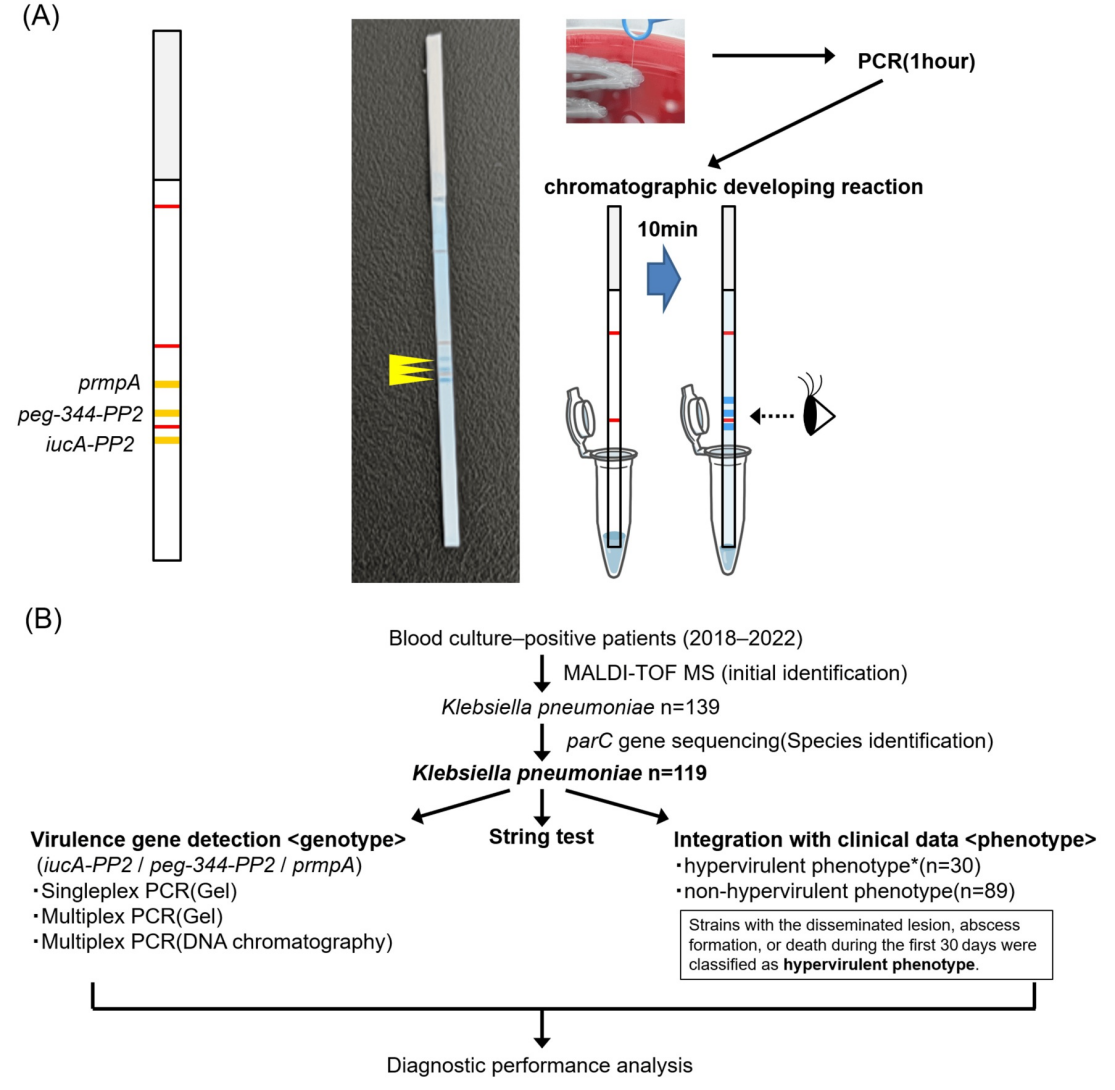
Schematic illustration of the DNA chromatography method and overview of the study workflow. (A) Schematic illustration of the DNA chromatography method and the chromatographic printed array strip used in this study. A schematic of the strip before use is shown on the left. As shown on the right, positive genes are confirmed as blue bands after the reaction. (B) Overview of the study workflow. A total of 119 strains identified as *Klebsiella pneumoniae *based on the parC gene sequence were analyzed. For genotypic analysis, three targeted virulence-associated genes (iucA-PP2, peg-344-PP2, and prmpA) were examined. The “hypervirulent phenotype” was determined based on clinical criteria.

DNA chromatography was performed using the single-tag hybridization (STH) chromatographic printed array strip (PAS) method, as previously described [11]. In this study, we used the C-PAS4 membrane (Sendai, Japan: TBA Co., Ltd.), which allows simultaneous visualization of four signals (Figure 1A). Specifically, PCR targeting virulence factor genes (iucA-PP2, peg-344-PP2, and prmpA) was performed under the conditions described above using primers labeled with both a tag and biotin. The PCR products were mixed with the developing and latex solutions included in the kit. The PAS was immersed in this mixture, and the chromatographic developing reaction was allowed to proceed for 10 min.

Clinical isolates

Clinical isolates were analyzed using an established multiplex PCR system targeting three virulence-associated genes (iucA-PP2, peg-344-PP2, and prmpA) for genotypic analysis. For comparison, singleplex PCR was also performed. An overview of the study is shown in Figure 1B. All patients with *K. pneumoniae* detected in blood samples who were admitted to Saitama Medical University Hospital between 2018 and 2022 were included in this study. Only the first episode of bacteremia in each patient was included in this retrospective analysis, and 139 patients were finally enrolled. All isolates derived from blood cultures were identified by matrix‑assisted laser desorption/ionization time-of-flight mass spectrometry (MALDI‑TOF MS) using a MALDI Biotyper version 3.1 and the MALDI Biotyper Reference Library version 4.0.0.1 (Bremen, Germany: Bruker Daltonics), according to the manufacturer’s instructions, and were stored at -80°C. Antimicrobial susceptibility testing was performed using a Microscan Walk Away 96 plus (Brea, CA: Beckman Coulter), and potential extended-spectrum beta-lactamase (ESBL) producers were further tested and confirmed using a combined disk test in accordance with the Clinical and Laboratory Standards Institute (CLSI) guidelines (https://clsi.org/) (document #M100-S27). The definition of the hypervirulent phenotype is the same as previously described.

Identification of* K. pneumoniae*

The isolates, which were initially identified as *K. pneumoniae* using MALDI-TOF MS and stored until use, were identified by sequence analysis of subunit C of the topoisomerase IV (parC) gene. Primers ParC-F (CTGAATGCCAGCGCCAAATT) and ParC-R (TGCGGTGGAATATCGGTCGC) were used, as previously described [5]. All isolates were cultured on blood agar in advance. To prepare the template DNA for PCR, bacterial suspensions adjusted to approximately 10^4^ CFU and heat-treated at 95°C for 5 min were used as the PCR template. Briefly, PCR amplicons were generated using TaKaRa SPPD STA (Kusatsu, Japan: Takara Bio Inc.), and thermal cycling was carried out under the following conditions: 98°C for 2 min, followed by 40 cycles at 98°C for 5 s, 55°C for 15 s, 72°C for 10 s, with a final extension at 72°C for 3 min. PCR products were analyzed using a 1% (w/v) agarose gel stained with ethidium bromide and purified using ExoSAP-IT Express PCR Product Cleanup Reagent (Waltham, MA: Thermo Fisher Scientific). Sanger sequencing of these purified PCR amplicons was performed by Eurofines Genomics Co., Ltd. (Tokyo, Japan). A phylogenetic analysis was performed using MEGA 11 (https://www.megasoftware.net/). The phylogenetic tree was constructed using the neighbor-joining method based on the analysis of 389 bp of the parC gene of 139 clinical isolates and reference strains. Klebsiella species were determined by identifying the cluster of reference strains to which they belonged in the phylogenetic tree.

The parC gene sequences of reference strains were obtained from the NCBI database (GenBank accession numbers: *K. pneumoniae*, AP006725, CP009208, CP026586, NZ_CP015134, NZ_CP030269, and NC_016845; *K. variicola*, NZ_CP017289, CP045783, and NZ_CP030173;* K. quasipneumonia*e, CP014696, and NZ_CP065838; *K. quasivariicola*, NZ_CP084768; and *K. africana*, NZ_CP084874). The reliability of the tree topology was checked using 500 bootstrap replicates.

Statistical analysis

Univariate analyses were conducted using Fisher’s exact test and unpaired Student's t-tests. To evaluate the diagnostic performance of hvKp strains based on target gene detection, a receiver operating characteristic (ROC) curve analysis was performed. To assess the diagnostic accuracy of DNA chromatography for target gene detection, 2×2 contingency tables were constructed using the conventional method (singleplex PCR and gel electrophoresis) as the reference standard. The agreement between the two assays was evaluated using overall agreement and Cohen’s kappa coefficient based on a contingency table. All statistical analyses were performed using EZR version 1.55 (Saitama, Japan). Statistical significance was set at p<0.05.

## Results

Accuracy evaluation

**Figure 2:**
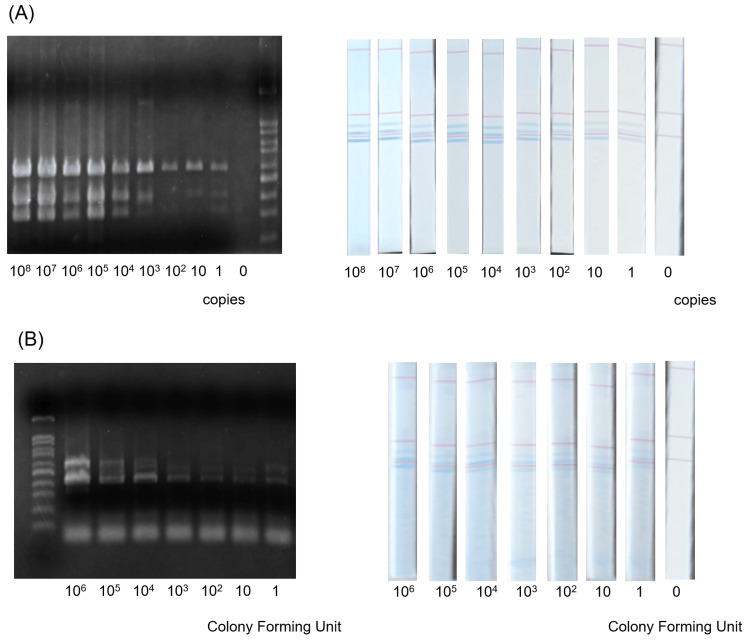
Analytical performance of multiplex PCR methods using gel electrophoresis and DNA chromatography. (A) Evaluation using synthetic artificial genes. The left side shows the results of gel electrophoresis, and the right side shows the results of DNA chromatography. (B) Evaluation using a strain in which all target genes were positive in a 1-CFU bacterial culture. The left side shows the results of gel electrophoresis, and the right side shows the results of DNA chromatography. CFUs: colony-forming units

First, using the multiplex PCR system we established, we compared the analytical performance of the conventional agarose gel electrophoresis method and DNA chromatography. Synthetic artificial genes were used for this evaluation. When the three genes were analyzed by singleplex PCR, prmpA was detectable at 10^4^ copies, iucA-PP2at 10^5^ copies, and peg-344-PP2 at 1 copy. When the three genes were analyzed simultaneously by multiplex PCR using agarose gel electrophoresis,prmpA was detectable at 10^6^ copies,iucA-PP2 at 10^4^ copies, and peg-344-PP2 at one copy (Figure 2A). Under the same conditions, when multiplex PCR followed by DNA chromatography was used, all three genes were detectable at 1 copy (Figure 2A). Next, using a strain in which all target genes were positive in 1 colony-forming unit (CFU) bacterial suspension, serial dilutions ranging from 106 CFU to 1 CFU were prepared, and the analytical performance was evaluated in the same manner. Both multiplex PCR with gel electrophoresis and multiplex PCR with DNA chromatography could detect all targets down to 1 CFU (Figure 2B).

Analysis of related genes and phenotypes of hypervirulent strains

**Figure 3:**
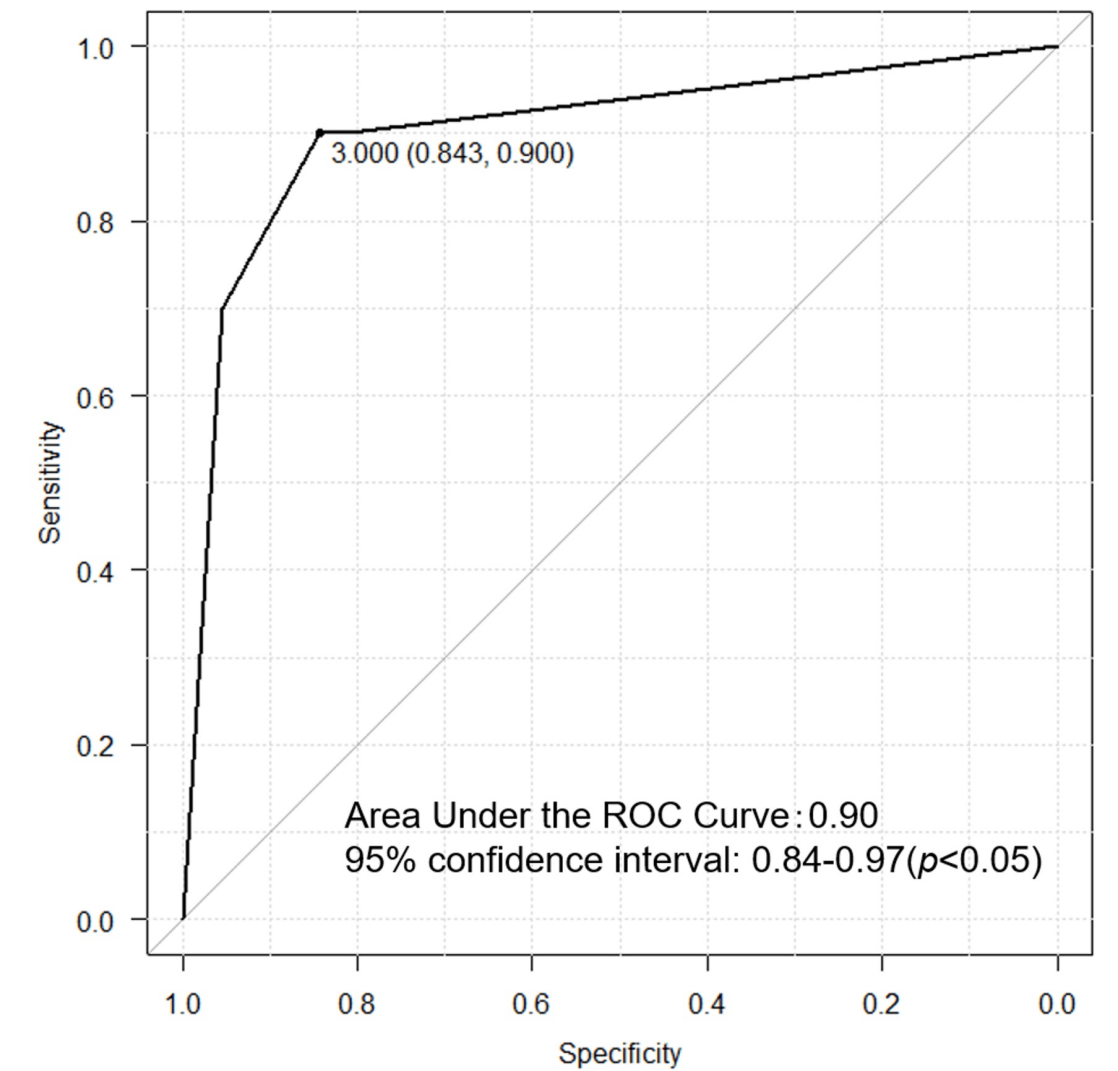
ROC curve for the diagnosis of hvKp based on singleplex PCR. The y-axis represents sensitivity, and the x-axis represents specificity. HvKp: hypervirulent *Klebsiella pneumoniae; *ROC​​​​​​: receiver operating characteristic; PCR: polymerase chain reaction

We evaluated the utility of multiplex PCR using gel electrophoresis and multiplex PCR using DNA chromatography with clinical isolates. Among the 139 isolates initially identified as *K. pneumoniae* by MALDI-TOF MS, the parC gene sequence analysis reclassified 119 isolates (85.6%) as* K. pneumoniae* and 20 (14.4%) as *K. quasipneumoniae* (figure in appendix). Thirty isolates were classified as having the hypervirulent phenotype based on the clinical course, and all were identified as *K. pneumoniae*. The prevalence of prmpA, iucA-PP2, and peg-344-PP2 (singleplex PCR) and the positivity rate of the string test were 90% (27 isolates), 60% (18 isolates), 90% (27 isolates), and 73% (22 isolates), respectively (Table 1). The prevalence of these genes was significantly higher in hypervirulent phenotype isolates (all p<0.01), and prmpA showed the highest sensitivity and specificity (odds ratio {OR}: 42.3, 95% confidence interval {CI}: 11.1-245,p<0.01) (Table 1). Using DNA chromatography, the positivity rates of prmpA, iucA-PP2, and peg-344-PP2 were 90% (OR: 42.3, 95% CI: 11.1-245), 87% (OR: 33.3, 95% CI: 9.61-152), and 90% (OR: 39.2, 95% CI: 10.3-226), respectively (Table 1). In the receiver operating characteristic (ROC) analysis, positivity for three or more markers among the four variables (string test and the three virulence-associated genes {iucA-PP2, peg-344-PP2, and prmpA}) yielded a sensitivity of 0.84 and a specificity of 0.90 (Figure 3). Among the 30 isolates classified as having the hypervirulent phenotype, three isolates were negative for all three genes and also negative in the string test. These cases showed no disseminated lesions or abscesses; delayed presentation, hypoglycemia, or underlying malignancy may have contributed to the cause of death. In other words, these were cases in which conditions other than *K. pneumoniae* may have contributed to mortality. Nevertheless, since all patients included in this study had *K. pneumoniae* bacteremia, the bacteremia itself may also have significantly contributed to death, and all cases that died within 30 days were uniformly classified as having the hypervirulent phenotype. All *K. quasipneumoniae* isolates were non-hypervirulent phenotypes and lacked the three target genes. Eight extended-spectrum beta-lactamase (ESBL)-producing strains were identified in both species, all of which exhibited a non-hypervirulent phenotype. None of the isolates examined in this study exhibited carbapenem resistance.

**Table 1:** Differences in bacterial characteristics between isolates of hypervirulent and non-hypervirulent phenotypes.

Variables		Hypervirulent phenotype (n=30)	Non-hypervirulent phenotype (n=89)	Sensitivity	Specificity	Positive predictive value	Negative predictive value	p-Value (Fisher’s exact test)	Odds ratio	95% confidence interval
String test		22	13	0.73	0.85	0.63	0.90	<0.01	15.5	5.36-50.0
prmpA	Singleplex PCR	27	15	0.90	0.83	0.64	0.96	<0.01	42.3	11.1-245
	Multiplex PCR	27	11	0.90	0.88	0.71	0.96	<0.01	59.9	15.1-356
	DNA chromatography	27	15	0.90	0.83	0.64	0.96	<0.01	42.3	11.1-245
iucA-PP2	Singleplex PCR	18	15	0.60	0.83	0.55	0.86	<0.01	7.24	2.69-20.5
	Multiplex PCR	26	11	0.87	0.88	0.70	0.95	<0.01	43.6	12.2-205
	DNA chromatography	26	14	0.87	0.84	0.65	0.95	<0.01	33.3	9.61-152
peg-344-PP2	Singleplex PCR	27	16	0.90	0.82	0.63	0.96	<0.01	39.2	10.3-226
	Multiplex PCR	27	13	0.90	0.85	0.68	0.96	<0.01	49.8	12.8-292
	DNA chromatography	27	16	0.90	0.82	0.63	0.96	<0.01	39.2	10.3-226

An analysis of 44 isolates that were positive for at least one marker by singleplex PCR demonstrated that some isolates that were negative in the string test were reclassified as hypervirulent based on gene detection (Table 2). Clinically, after excluding 20 patients with* K. quasipneumoniae* bacteremia, diabetes mellitus was the most common comorbidity. Hospital-acquired infection occurred more frequently in the non-hypervirulent phenotype group, whereas pneumonia and abdominal infections (including liver abscesses) were significantly more common sources of bacteremia in hypervirulent phenotype cases (appendix 2).

**Table 2:** The results of the 44 strains that were positive for any of the string test, prmpA, iucA-PP2, and peg-344-PP2. Among the 30 isolates classified as the hypervirulent phenotype, three were negative for the string test as well as all three genes. Therefore, 27 of the 44 isolates that were positive for any of the string tests, prmpA, iucA-PP2, or peg-344-PP2 were classified as the hypervirulent phenotype. +: positive; -: negative

Patient number	Classified as hypervirulent phenotype	String test	iucA-PP2	peg-344-PP2	prmpA
1	No	-	+	+	+
2	No	-	+	-	-
3	No	-	-	+	+
4	No	-	+	+	+
5	No	+	-	+	+
6	No	+	+	+	+
7	No	+	-	+	+
8	No	+	+	+	+
9	No	+	-	+	-
10	No	+	+	+	+
11	No	+	+	+	+
12	No	+	-	+	+
13	No	+	-	+	+
14	No	+	-	+	+
15	No	+	-	+	+
16	No	+	-	+	+
17	No	+	-	+	+
18	Yes	-	+	+	+
19	Yes	-	+	+	+
20	Yes	-	+	+	+
21	Yes	-	+	+	+
22	Yes	-	+	+	+
23	Yes	+	+	+	+
24	Yes	+	+	+	+
25	Yes	+	+	+	+
26	Yes	+	+	+	+
27	Yes	+	+	+	+
28	Yes	+	+	+	+
29	Yes	+	+	+	+
30	Yes	+	+	+	+
31	Yes	+	+	+	+
32	Yes	+	+	+	+
33	Yes	+	+	+	+
34	Yes	+	+	+	+
35	Yes	+	+	+	+
36	Yes	+	+	+	+
37	Yes	+	+	+	+
38	Yes	+	+	+	+
39	Yes	+	+	+	+
40	Yes	+	+	+	+
41	Yes	+	+	+	+
42	Yes	+	+	+	+
43	Yes	+	-	+	+
44	Yes	+	+	+	+

Concordance of multiplex PCR methods (gel electrophoresis and DNA chromatography) with singleplex PCR

The concordance between multiplex PCR (gel electrophoresis and DNA chromatography) and singleplex PCR was evaluated using clinical isolates. The results of multiplex PCR (gel electrophoresis) and multiplex PCR (DNA chromatography) were compared with the singleplex PCR results as the reference standard. In the comparison between singleplex PCR and multiplex PCR (gel electrophoresis), the overall agreement between the two assays was 96.6% and 97.4%. Cohen’s κ coefficients ranged from 0.92 to 0.95, indicating almost perfect agreement. In the comparison between singleplex PCR and multiplex PCR (DNA chromatography), the overall agreement between the two assays was 94.1% and 100%. Cohen’s κ coefficients ranged from 0.86 to 1.00, also indicating almost perfect agreement (Table 3).

**Table 3:** Concordance between singleplex PCR and multiplex PCR (gel electrophoresis or DNA chromatography). PCR: polymerase chain reaction

Gene	Comparison	Singleplex positive- multiplex positive	Singleplex negative- multiplex positive	Singleplex positive- multiplex negative	Singleplex negative- multiplex negative	Overall agreement	Cohen's kappa coefficient
prmpA	Multiplex PCR (gel electrophoresis) versus singleplex PCR	38	0	4	77	96.6% (115/119)	0.93
	Multiplex PCR (DNA chromatography) versus singleplex PCR	42	0	0	77	100% (119/119)	1.00
iucA-PP2	Multiplex PCR (Gel electrophoresis) versus singleplex PCR	33	4	0	82	96.6% (115/119)	0.92
	Multiplex PCR (DNA chromatography) versus singleplex PCR	33	7	0	79	94.1% (112/119)	0.86
peg-344-PP2	Multiplex PCR (Gel electrophoresis) versus singleplex PCR	40	0	3	76	97.5% (116/119)	0.95
	Multiplex PCR (DNA chromatography) versus singleplex PCR	43	0	0	76	100% (119/119)	1.00

## Discussion

In this study, we attempted to improve the diagnostic accuracy for hvKp strains by combining the conventional string test with the detection of virulence genes previously reported to be specific for hvKp strains (prmpA, iucA-PP2, and peg-344-PP2) [5]. A novel aspect of this study is the establishment of a multiplex PCR and nucleic acid chromatography system that enables rapid gene detection directly from bacterial colonies. Conventional methods require gel electrophoresis and staining procedures, which are time-consuming and require technical expertise. In contrast, our method enables the rapid and straightforward determination of the presence or absence of hvKp-associated genes, with results obtainable within approximately one hour, suggesting high practical utility in clinical settings.

Although the string test has been widely used as a phenotypic marker of hvKp strains, its sensitivity and specificity are not necessarily high [12]. Severe hvKp infections have been reported in string test-negative cases. In the present study, we also identified cases that were string test-negative but classified into the hypervirulent phenotype group, suggesting that reliance solely on phenotypic methods may lead to underdiagnosis of hvKp infections. In addition, consistent with previous reports, the three targeted virulence-associated genes also demonstrated high sensitivity and specificity in this study. Therefore, the combined use of genotypic markers may be helpful for the diagnosis and potential risk stratification of hvKp infections. Disseminated lesions and metastatic infections are characteristic features of the hypervirulent phenotype; however, these complications may not become clinically apparent until some time after the onset of bacteremia. Therefore, in severe cases or when the hypervirulent phenotype is clinically suspected, early gene testing may support consideration of early imaging evaluation for potential metastatic foci and appropriate source control. A rapid genotypic diagnosis may thus contribute not only to microbiological identification but also to overall clinical management.

The multiplex PCR system established in this study could detect as few as 10^6^ copies using synthetic artificial genes and one colony-forming unit (CFU) when evaluated with a 1-CFU bacterial suspension. Given that the bacterial load in severe bacteremia has been reported to be approximately 100 CFU, the analytical sensitivity of our method appears sufficient for clinical application [13]. Furthermore, a high concordance rate was observed between nucleic acid chromatography and conventional gel electrophoresis, supporting the reliability of the assay. Because nucleic acid chromatography does not require electrophoresis or staining, results can be obtained rapidly after multiplex PCR, representing a major advantage as a rapid diagnostic method.

Regarding the clinical background, consistent with previous reports, significantly fewer cases in the hypervirulent phenotype group met the definition of hospital-acquired infection, and most were considered community-acquired. In addition, similar to previous reports, patients in the hypervirulent phenotype group more frequently presented with pneumonia and disseminated lesions, particularly liver abscesses [6,7]. Death within 30 days was also more common in the hypervirulent phenotype group; however, this may partly reflect the inclusion of “death within 30 days” in the definition of the hypervirulent phenotype. Nevertheless, there was no significant difference between the two groups in terms of appropriate antibiotic therapy within 24 h, indicating that appropriate treatment was initiated early in many cases. Considering this, our findings are consistent with previous reports, suggesting that the hypervirulent phenotype was associated with a higher risk of mortality [7]. In contrast, although diabetes mellitus has traditionally been regarded as an important risk factor for the hypervirulent phenotype, no significant difference was observed between the hypervirulent and non-hypervirulent phenotype groups in this study. This finding suggests that host factors alone may not reliably predict hvKp infection and underscores the importance of molecular diagnostic approaches.

This study has several limitations. First, as it was conducted at a single center with a small sample size, the results may have been influenced by region-specific circulating strains and may not necessarily be reproducible in other institutions within the same country or in other countries; therefore, validation through multicenter studies is warranted. Second, we did not analyze capsular genotypes or sequence types (STs) characteristic of hvKp strains (e.g., K1-ST23 and K2-ST65), nor did we perform plasmid typing, as this study focused primarily on the detection of virulence-associated genes [14]. Moreover, other hvKp-associated genes have been reported, and the diagnostic performance may vary depending on the selected gene targets; thus, expansion of the gene panel should be considered in future studies. Although the present study established a colony-based detection system, the development of a method capable of directly detecting virulence genes from positive blood culture bottles would enable even more rapid diagnosis. Isothermal amplification methods, such as loop-mediated isothermal amplification (LAMP), may also represent a promising approach. The development of rapid, simple, and highly sensitive diagnostic tools for hvKp remains an important future challenge.

## Conclusions

In conclusion, although this study was conducted at a single center with a limited sample size, the rapid multiplex PCR combined with nucleic acid chromatography established in this study enables the simultaneous detection of previously reported hvKp-associated virulence genes in a simple and timely manner. This method may help facilitate the identification of hypervirulent* K. pneumoniae* and support consideration of clinical risk assessment in patients with suspected hvKp infections. This approach may contribute to future advances in the molecular epidemiology of hvKp and potential improvements in clinical management.

## Figures and Tables

**Figure 4:**
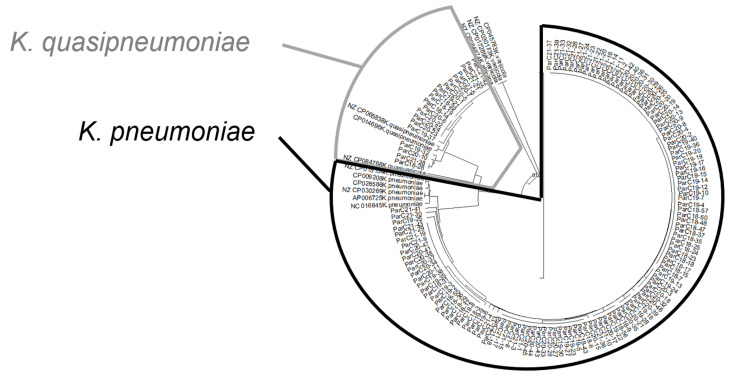
Phylogenetic tree of the 139 clinical isolates and reference strains used in this study. Isolates identified as* Klebsiella pneumoniae *are indicated by black boxes, whereas those identified as *Klebsiella quasipneumoniae* are indicated by gray boxes. This image is created by the authors of this study using MEGA 11 (https://www.megasoftware.net/) and Microsoft PowerPoint.

## Appendices

Appendix 1

**Table 4:** Synthetic artificial genes.

Gene name	Synthetic gene sequence	Gene size (including restriction enzyme sites)	Vector backbone	Restriction enzyme site	
prmpA	GAGTAGTTAATAAATCAATAGCAATTAAGCACAAAAGAAACATAAGAGTATTGGTTGACAGCAGGATTTTTTATTCAGGGAAATGGGGAGGGTACAAAATGTTAAGGGGATCATTAAATATGATAAGCCAATGGATGTGGCTTGACGTTTCGGGGGGGGGGCGGTTTTATCCTAAAGGGTGTGATTATGACATCTATGTTAACATGCAAGGAAATGTAAAAAATAATATTGAAAAACTATATTTTGCATTCTTAAAGAAAAATGTTAGCCGAATTGTAAACCATTATCCACGGCTAACAAAAAAGGAACAAGCAGTGCTGCAATGCCTACTG	344 bp	pEX-A2J2	5'EcoRI	3'XhoI
iucA-PP2	TCAGCCCTTTAGCGACAAGCTCCTGGCACCATTCCTGCTGCAACAGATACTCCCCCTGCCACGGGTGCAGCGGGAATAGCCATTGATTGTCGCTTAATTCATTGAGTAACTGCGGCGCATTCTCTGCGGCAAATCGCGTCAACCGTTGTTGCAGGTTGAGATGCAGGCTTTCACCGGCGATCTGCGTTTTGTTCACCGCAAACCACCGCAGCGGGAAGTGGGGCGCGAAGTCCGGCAGGTAGCGTTCGGCCTCCTGCTGGTTAAACGGTTCATGAGACTTAGGCGCCGGGTGGAAGGCGTGCCCGACCAGCAGAGCCTGCTCCGCCTCGCCAAAGTTCAACGCTTTTTCACGCAGGGCTGCCCAGTCATGGCGGGCATCAATTGCCTGCTGCGTATGGGCGTGGCTTTCCATCACGCGCTGGTGGAAGGTTTCGCAACTGGTCGCGGGTAGCAAGAGTTGGTGCTGGAGCTTATCAACAATAAGCCGGGAGAGGGTGGCGAAATCGACGGGGTGACTGCCGGAGGCCGTGACCAGACGCGCCGGAAATTCAAAGCGGTGATGCTGGGTTGGGGAGAAATAAGC	565 bp	pEX-A2J2	5'EcoRI	3'XhoI
peg-344-PP2	AAAGGACAGAAAGCCAGTGGACGGGAGTTATTGCCCTTTCTTCTGCAGTAATAATTCATGCCATAGTATATACTCAATGTAAAAAAAGATGCTGTAAAGTCTCTGTTATATCGTTTAATGCGTTACCGTGTTTTATAGCTGGGGTTATTCTTTCGCTGGTGGGGAGTATCTTTGAGAGGCCACAATTATCAGCTTTATCTTTACACTCTACATTAGCAACAATGTATTTAGGCTGTTTTGCTGGAGTTTTTGGGATACTGTGCTATTTTTCTCTGCAGAAAAGGGCTAGCGCTTTCCAGGCTTCGCTCGTATTTCTCATCTTCCCTCTAATTGCAGTAAGCTTGGAAAGTTATATATATGGGAAAACCATATCAACATACTCAATCTTACTGATTATCCCCCTCGTCATTG	423 bp	pEX-A2J2	5'EcoRI	3'XhoI

Appendix 2

**Table 5:** Differences in clinical characteristics between patients with hypervirulent phenotype and non-hypervirulent phenotype Klebsiella pneumoniae bloodstream infections. †Fisher’s exact test. ‡Student's t-test. *Hospital-acquired infection was defined as an infection occurring ≥48 h after hospital admission. Data are presented as the number (percentage) or as the median (interquartile range) unless otherwise specified. ADL: activities of daily living; CRBSI: catheter related blood stream infection; IE: infectious endocarditis

Characteristics of patients		Total (n=119)	Hypervirulent phenotype (n=30)	Non-hypervirulent phenotype (n=89)	p-Value
Age, median, years		72 (64-79)	72 (63-79)	72 (66-82)	0.25‡
Age >80 years		29 (24.4)	10 (33.3)	19 (21.4)	0.22†
Male sex		64 (53.8)	17 (56.7)	47 (52.8)	0.83†
Comorbidities	Diabetes mellitus	34 (28.6)	10 (33.3)	24 (27.0)	0.49†
	Malignancy	24 (20.2)	7 (23.3)	17 (19.1)	0.61†
	Liver cirrhosis	11 (9.24)	3 (10.0)	8 (8.99)	1.00†
	Collagen disease	11 (9.24)	1 (3.33)	10 (11.2)	0.29†
	Chronic kidney disease	19 (16.0)	3 (10.0)	16 (18.0)	0.40†
	Pulmonary disease	13 (10.9)	1 (3.33)	12 (13.5)	0.18†
	Mental disorder	6 (5.04)	3 (10.0)	3 (3.37)	0.17†
	Gastrointestinal disease	8 (6.72)	1 (3.33)	7 (7.87)	0.68†
	Disease of pancreas, disease of liver-biliary tract system	26 (21.8)	6 (20.0)	20 (22.5)	1.00†
	Hematologic diseases	14 (11.8)	3 (10.0)	11 (12.4)	1.00†
Immunosuppressive drug		15 (12.6)	3 (10.0)	12 (13.5)	0.76†
Neutropenia		5 (4.20)	1 (3.33)	4 (4.49)	1.00†
Hospital-acquired infection*		42 (35.3)	5 (16.7)	37 (41.6)	0.02†
Appropriate antibiotic therapy within 24 h		112 (94.1)	27 (90.0)	85 (95.5)	0.37†
Source of infection	Pneumonia	26 (21.8)	15 (50.0)	11 (12.4)	<0.001†
	Skin and soft tissue	3 (2.52)	2 (6.67)	1 (1.12)	0.16†
	Abdominal (including liver abscess)	22 (18.5)	12 (40.0)	10 (11.2)	<0.001†
	Liver abscess	7 (5.88)	7 (23.3)	0 (0.00)	<0.001†
	Urinary tract	35 (29.4)	9 (30.0)	26 (29.2)	1.00†
	Meningitis	1 (0.84)	1 (3.33)	0 (0.00)	0.25†
	Endophthalmitis	2 (1.68)	2 (6.67)	0 (0.00)	0.06†
	Others	37 (31.1)	4 (13.3)	33 (37.1)	0.02†
Death during the first 30 days		12 (10.1)	12 (40.0)	0 (0.00)	<0.001†
Disseminated lesion		9 (7.56)	9 (30.0)	0 (0.00)	<0.001†
Abscess formation (including liver abscess)		18 (15.1)	18 (60.0)	0 (0.00)	<0.001†
